# Construction of Composite Biocontrol Agent (BCA): Developing Effective Strategies for Controlling Postharvest Blue Mold and Patulin in Apples

**DOI:** 10.3390/foods14193378

**Published:** 2025-09-29

**Authors:** Longmei Cong, Limei Li, Qian Zhang, Junyue Hu, Jingting Du, Junfeng Shi

**Affiliations:** College of Food Science and Engineering, Shanxi Agricultural University, Taiyuan 030031, China; z20223635@stu.sxau.edu.cn (L.C.); 18434602993@163.com (L.L.); celiazhq@126.com (Q.Z.); z20223645@stu.sxau.edu.cn (J.H.); djtbxs@126.com (J.D.)

**Keywords:** apple postharvest disease, *Penicillium expansum*, composite biocontrol agent (BCA), Patulin (PAT) degradation, Response Surface Methodology (RSM)

## Abstract

Postharvest blue mold in apples, caused by *Penicillium expansum*, leads to fruit decay and patulin (PAT) contamination, incurring major economic and health risks. This study developed a composite biocontrol agent (BCA) by co-cultivating three antagonistic yeasts (*Meyerozyma caribbica*, *Metschnikowia zizyphicola*, and *Pichia rarassimilans*). Mixed-culture conditions and protective additives formulation were optimized via response surface methodology. Optimal biomass production was achieved with a 1:2:3 (*v*/*v*/*v*) yeast ratio in medium containing sucrose (12.49 g/L), yeast extract powder (13.3 g/L), K_2_HPO_4_ (0.88 g/L), and NaCl (0.95 g/L) under pH 7.0, 1% total inoculum concentration, 24 °C, and a 60 h incubation. The liquid BCA formulation, stabilized with 0.27% gum arabic, 0.49% Tween-80, and 0.079% ascorbic acid, maintained high viability (9.15 log10 CFU/mL after 7 days). In vivo/in vitro trials all demonstrated that the composite BCA rapidly colonized, suppressed *P. expansum* infection, and significantly delayed pathogen spore germination and hyphal growth. Furthermore, the BCA effectively degraded 10 μg/mL PAT within 24–42 h in various fruit juices with minimal adverse effects on juice quality parameters. Storage at −20 °C preserved the highest bioactivity (7.93 × 10^8^ CFU/mL after 5 months). This optimized composite yeast formulation provides an efficient, eco-friendly strategy for integrated apple postharvest blue mold and PAT detoxification.

## 1. Introduction

Postharvest blue mold decay in apples, primarily caused by *Penicillium expansum*, is one of the most prevalent diseases affecting apples during harvesting, transportation, and storage, mainly controlled through synthetic fungicides in practice. *P. expansum* is characterized by its strong reproductive capacity, diverse infection pathways, and high environmental adaptability. Its invasion not only causes fruit flesh decay, leading to loss of commercial value, but also produces the harmful secondary metabolite patulin (PAT). PAT accumulates in the decayed areas of the fruit and rapidly diffuses into sound tissues. It can subsequently enter processed products such as juices and jams during processing [1,2]. Severe contamination can cause acute, subacute, and chronic toxicity in humans and animals [3,4]. Existing physical or chemical techniques struggle to effectively suppress the disease and remove the toxin without compromising the quality of apples and their products [5,6], which severely hinders the healthy development of the apple industry.

Compared to the residue issues associated with chemical fungicides and the impact of physical methods on fruit quality, biocontrol technology offers a safer and more effective alternative strategy due to its eco-friendliness, health safety, and mild reaction conditions [7]. Among various biocontrol microorganisms, yeasts stand out and are widely employed in postharvest preservation research and application for fruits and vegetables. They are favored over bacteria and molds due to their broad availability, rapid adaptability and colonization, safety for fruit application, and effective antagonistic activity [8,9,10]. They have been proven effective in controlling various postharvest pathogenic fungi, including *Botrytis cinerea*, *Alternaria alternata*, *P. digitatum*, *P. italicum*, *P. expansum*, *Rhizopus stolonifer*, *Colletotrichum* spp., and *Aspergillus niger* [2,11].

Beyond suppressing pathogenic fungi, antagonistic yeasts have proven effective against various mycotoxins. Researchers have conducted targeted screening of antagonistic yeasts for toxin degradation produced by various pathogens, identifying potent strains including *Rhodotorula mucilaginosa* [12], *Saccharomyces cerevisiae* [13], *Pichia caribbica* [14], and *Kluyveromyces marxianus* [15]. Preliminary mechanistic studies revealed strain-dependent divergence in biodetoxification pathways and degradation metabolites. Therefore, targeted research is necessary for different fruit varieties and yeasts in biocontrol strategies.

In practical applications, single antagonistic yeast agents are often constrained by limited antifungal efficacy and unstable performance, typically achieving inhibition rates of only 40% to 50%. In contrast, composite biocontrol agents (BCAs) have garnered widespread attention due to their ability to exert synergistic effects and enhance overall antifungal efficiency [16]. This approach can reduce the dosage and application frequency of BCAs while inhibiting the development of resistance in harmful microorganisms. Research by Janisiewicz et al. demonstrated that compared to equivalent amounts of single yeast species, a combination of *Metschnikowia pulcherrima* and *Cryptococcus laurentii* provided better control of apple postharvest decay caused by *Penicillium* [17]. Similarly, the combined application of three antagonistic yeasts (*Hanseniaspora uvarum*, *Meyerozyma guilliermondii*, and *Metschnikowia* aff. *Pulcherrima* P01A016) significantly inhibited green mold decay on citrus fruits. Both in vitro and in vivo trials confirmed synergistic cooperation among these yeasts, resulting in higher biocontrol efficacy than single-strain treatments [18]. Feng, M. et al. also found that a combination of *Cryptococcus albidus* (Ca63) and *Cryptococcus albidus* (Ca64) achieved a 75% preventive effect against gray mold on snap beans [16]. Furthermore, the combined use of *Pichia guilliermondii* and *Bacillus mycoides* under varying temperature and humidity conditions increased the inhibition rate of *Botrytis cinerea* on strawberry leaves and plants to 88.0–99.8% [19]. Currently, combining different microorganisms based on diverse biocontrol mechanisms represents a new research direction in the application of biocontrol for fruits. However, technical bottlenecks remain regarding the rapid proliferation and stability of viable cells in composite BCAs.

This study is based on three yeast strains (*Meyerozyma caribbica*, *Metschnikowia zizyphicola*, *Pichia rarassimilans*) previously isolated by our research group. These strains were combined to form a new composite BCA. Dual response surface methodology was employed to investigate the interactive effects of mixed culture conditions and key protective agents. The biocontrol efficacy of the BCA against apple blue mold and its ability to degrade patulin (PAT) in different fruit juices were systematically evaluated. The findings aim to provide theoretical foundation and technical support for the green control of postharvest blue mold in apples and mycotoxin management.

## 2. Materials and Methods

### 2.1. Experimental Materials

Biocontrol yeasts: Three antagonistic yeast strains—*M. caribbica* (Accession No. 27604), *P. rarassimilans* (Accession No. 32875), and *M. zizyphicola* (Accession No. M2016564)—were used in this study. *M. caribbica* and *P. rarassimilans* were preserved at the China General Microbiological Culture Collection Center, while *M. zizyphicola* was preserved at the China Center for Type Culture Collection. All strains had been previously isolated and purified by our research group from the surfaces of flat peach, grape, and apple, respectively. Prior to use, each strain was revived from 30% glycerol stocks stored at −80 °C, which had been preserved for less than six months.

Pathogenic fungus: *Penicillium expansum* was isolated and purified from naturally infected apple fruit by our research group. It was preserved using the same long-term cryopreservation method as the yeast strains (stored in 30% glycerol at −80 °C) and subsequently kept at 4 °C for ready use in subsequent experiments.

Fruit samples: Apples (cv. ‘Gala’) free from pests, diseases, and mechanical damage, with uniform maturity and size, were selected and stored at 0 °C until use.

Chemicals and reagents: Beef extract, yeast extract paste, glucose, agar powder, yeast extract powder, peptone, glucose, NaCl, and other chemicals used were of analytical grade. The patulin (PAT) standard and methanol used for high-performance liquid chromatography (HPLC) were of chromatographic grade and were purchased from Shanghai Macklin Biochemical Technology Co., Ltd. (Shanghai, China).

The patulin content was determined using HPLC. The system used was an Agilent 1260 system (Agilent Technologies, Santa Clara, CA, USA) with the Zorbax SB-C18 column (Agilent). The mobile phase consisted of water–acetonitrile (90:10, *v*/*v*) delivered at a flow rate of 1 mL/min under isocratic elution. Detection was carried out at a wavelength of 276 nm.

### 2.2. Experimental Methods

#### 2.2.1. Biocompatibility Assessment Among Yeast Strains

The cross-compatibility among the three yeast strains was assessed. Cell suspensions of each yeast strain were prepared and adjusted to a concentration of 1 × 10^8^ CFU/mL. Aliquots (100 μL) of each suspension were spread evenly onto Nutrient Yeast Dextrose Agar (NYDA) plates, serving as the indicator lawn. Subsequently, the same cell suspensions were concentrated to 1 × 10^9^ CFU/mL, and aliquots (10 μL) of each high-density suspension (test strains) were spotted at three distinct points, 2.5 cm apart from the center, on the plates seeded with the indicator lawn. Plates were incubated at 28 °C for 48 h and examined for inhibition zones between the indicator lawn and the spotted strains.

#### 2.2.2. Efficacy of Multi-Strain Yeast Cultures Against Apple Blue Mold

The three yeast strains (*M. caribbica*, *M. zizyphicola*, *P. rarassimilans*) were activated and individually inoculated into fresh Nutrient Yeast Dextrose Broth (NYDB). After 24 h of incubation at 28 °C and 180 rpm, single-strain seed cultures were obtained. Combined seed cultures were prepared by mixing the single-strain cultures at volume ratios of 1:1:1, 1:2:2, and 1:2:3. The total inoculation volume was 2% (*v*/*v*). Combined cultures were incubated for an additional 48 h, then centrifuged to harvest the cells.

Mature apples without mechanical damage were selected according to the method described by Shi et al. [20] with modifications. Fruits were surface-sterilized by immersion in 1.0% NaClO for 1 min, air-dried, and wounded at the equator using a sterile cork borer (4 wounds per fruit; 4 mm diameter, 5 mm deep). Each wound was treated with 50 μL of either single-strain or combined yeast culture fermentation broth, with sterile distilled water as the control. After air-drying, 20 µL of *P. expansum* spore suspension (1 × 10^4^ spores/mL) was challenged into each wound. Fruits were incubated at 25 °C under high humidity conditions. After 6 days of incubation, the lesion diameter and disease incidence were recorded, and the inhibition rate was calculated.

#### 2.2.3. Optimization of Nutrient Conditions for Mixed Culture

The mixed seed culture, prepared using the optimal ratio determined in Section 2.2.2, was employed for medium optimization. Yeast nitrogen base without amino acids (YNB) served as the basal nitrogen source. The effects of various nutritional factors on the viable cell count of the mixed culture were evaluated: carbon sources (glucose, molasses, lactose, sucrose, dextrin, and soluble starch) and the concentration gradients; nitrogen sources (yeast extract powder, yeast peptone, beef extract, tryptone, (NH_4_)_2_SO_4_, NH_4_Cl, and urea) and the concentration gradients; and inorganic salts (KH_2_PO_4_, K_2_HPO_4_, MnSO_4_, MgSO_4_, ZnSO_4_, NaCl, and K_2_SO_4_) and the concentration gradients. The viable cell count was used as the evaluation index.

Based on the results of the single-factor experiments, a four-factor, three-level Box–Behnken response surface methodology (RSM) design was employed to optimize the medium composition. Factors included Carbon source (A), Nitrogen source (B), K_2_HPO_4_ (C), and NaCl (D) concentrations, with viable cell count as the response value. The optimal medium composition was determined accordingly, and experimentally validated.

#### 2.2.4. Optimization of Fermentation Conditions for Mixed Culture

The mixed seed culture was inoculated into the optimized medium from Section 2.2.3. The effects of pH (5.0–9.0), total inoculation volume (0.5–4% *v*/*v*), incubation temperature (20–36 °C), and incubation time (24–72 h) on the viable cell count were investigated. The viable cell count served as the evaluation index.

#### 2.2.5. Optimization of Protective Additives Formulation for the Biocontrol Agent (BCA)

To enhance the stability and shelf-life of the liquid BCA, protective additives were incorporated. Equal volumes (1:1, *v*/*v*) of the mixed yeast suspension and the protective additives solution (containing varying concentrations of thickeners, emulsifiers, and antioxidants) were blended. The viable cell count after 7 days of storage was used as the evaluation criterion for single-factor optimization, with three replicates per treatment. Thickeners: 0.3% and 0.6% gum arabic, sodium alginate, or agar; Emulsifiers: 0.2% and 0.4% Tween-20 or Tween-80; Antioxidants: 0.03% and 0.06% ascorbic acid, tea polyphenols, or butylated hydroxytoluene (BHT).

Based on the single-factor results, a three-factor, three-level Box–Behnken RSM design was employed, using the viable cell count as the response variable to optimize the protectant formulation, with three replicates per run.

#### 2.2.6. Evaluation of Biocontrol Efficacy of the Composite BCA

A. Population Dynamics in Apple Wounds: The colonization ability of the composite BCA on apple wounds was assessed using a modified method as described by Lin et al. [21]. Apples were wounded and treated as described in Section 2.2.2. Each wound was inoculated with 50 μL of the liquid BCA suspension (1 × 10^8^ CFU/mL). After air-drying, fruits were stored at 20 °C under high humidity. Fruit tissue surrounding the wounds was sampled at intervals, homogenized, serially diluted with sterile distilled water, and plated to determine viable cell counts.

B. In Vivo Biocontrol Efficacy on Apple Fruit: Apples were treated as described in Section 2.2.2. Each wound was inoculated with 50 μL of the liquid BCA suspension (1 × 10^8^ CFU/mL), air-dried, and then challenged with 20 μL of the *P. expansum* spore suspension (1 × 10^4^ spores/mL). Fruits were incubated at 25 °C under high humidity for 6 days. Lesion diameter and disease incidence were measured to calculate inhibition rate for blue mold control efficacy assessment.

C. In Vitro Antifungal Efficacy: The direct inhibitory effect of the BCA against *P. expansum* growth was assessed using a modified method as described by Wu et al. [22]. Sterile Petri dishes containing 50 mL PDA were prepared, and a central well (5 mm diameter) was created using a sterile cork borer. Each well received 50 μL of BCA suspension (1 × 10^8^ CFU/mL) or sterile distilled water (control). After air-drying, wells were challenged with 20 μL of the *P. expansum* spore suspension (1 × 10^4^ spores/mL). Plates were incubated at 25 °C for 6 days, after which the colony diameter of *P. expansum* was measured to calculate inhibition rates.

D. Scanning Electron Microscopy (SEM) Observation: Apples were wounded and treated as described in Section 2.2.2. (BCA application followed by *P. expansum* challenge). At 24 h and 48 h post inoculation, fruit tissues from the wound site were excised with sterile blades. Samples underwent fixation in glutaraldehyde at 4 °C overnight, sequential ethanol dehydration, critical-point drying, gold sputter-coating, and examination with a Hitachi SU8010 SEM to characterize yeast-pathogen interfacial interactions on wounds, according to the protocol of Pietrysiak et al. [23].

#### 2.2.7. Degradation of PAT in Different Fruit Juices by the Composite BCA and Impact on Juice Quality

The ability of the composite BCA to degrade PAT in various juices was assessed according to Zheng et al. [24]. Aliquots (1 mL) of the composite BCA suspension (1 × 10^8^ CFU/mL) were added to 50 mL of different fruit juices (orange, pear, apple, grape). An equal volume of sterile distilled water served as the blank control. PAT stock solution was then added to obtain a final concentration of 10 μg/mL. All the samples were incubated with shaking. At 6 h intervals, aliquots were withdrawn, centrifuged, filtered (0.22 μm membrane), and analyzed for PAT content using HPLC.

The impact of BCA addition on juice quality parameters was evaluated as described by Liu et al. [25]. The pH, soluble solids content (SSC), non-enzymatic browning index (NEBI), and light transmittance were measured in both BCA-treated and control juices after the incubation period.

#### 2.2.8. Storage Stability of the Liquid Composite BCA

The formulated liquid BCA was stored at three temperatures: 25 °C, 4 °C, and −20 °C. The viable cell count was monitored periodically throughout the storage period.

### 2.3. Statistical Analysis

Each experiment had three biological replicates (*n* = 3). All data were analyzed by one-way ANOVA with Duncan’s post hoc test for multiple comparisons using SPSS 22.0, after verifying normality (Shapiro–Wilk test) and homogeneity of variances (Levene’s test) assumptions (*p* > 0.05). Pearson’s correlation analysis was applied where appropriate, with data meeting bivariate normality. Statistical significance was defined at *p* < 0.05 for all tests.

## 3. Results

### 3.1. Biocompatibility Assessment Among Yeast Strains

**Figure 1:**
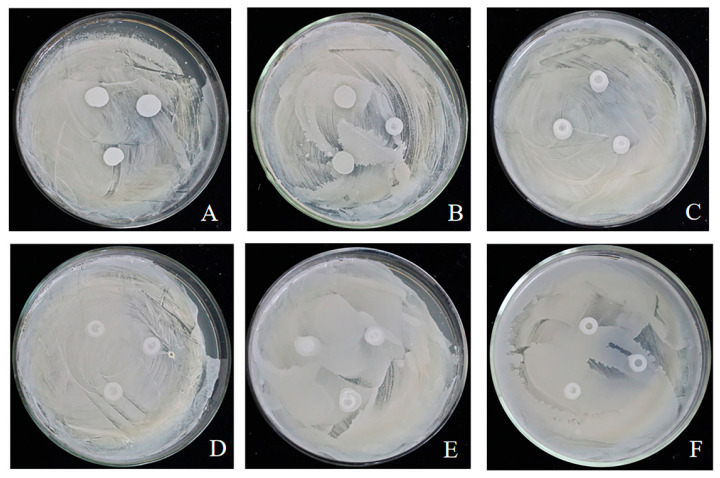
Assessment of cross-compatibility among yeast strains. Note: (**A**–**F**) Dual-culture assays showing the reciprocal interactions between each pair of strains. Each panel represents a different combination where the lawn culture (indicator strain) is challenged with a spot-inoculated test strain (as detailed below). (**A**) *M. caribbica* (lawn) vs. *M. zizyphicola* (test spot). (**B**) *M. caribbica* (lawn) vs. *P. rarassimilans* (test spot). (**C**) *M. zizyphicola* (lawn) vs. *M. caribbica* (test spot). (**D**) *M. zizyphicola* (lawn) vs. *P. rarassimilans* (test spot). (**E**) *P. rarassimilans* (lawn) vs. *M. caribbica* (test spot). (**F**) *P. rarassimilans* (lawn) vs. *M. zizyphicola* (test spot).

The presence or absence of inhibition zones around the spots of the test strains was observed. An inhibition zone indicated antagonism between strains, while its absence indicated no inhibitory effect on mutual growth. The three yeast strains (*M. caribbica*, *M. zizyphicola*, *P. rarassimilans*) were reciprocally cross-tested for biocompatibility, with each strain serving as both the lawn culture and the test strain in a reciprocal manner. As shown in Figure 1, no inhibition zones were observed around any test strain spots after 48 h incubation at 28 °C under standard conditions, irrespective of strain combinations. These results confirm mutual compatibility without detectable antagonistic interactions, validating their potential for development as a multi-strain biocontrol agent.

### 3.2. Efficacy of Multi-Strain Yeast Cultures Against Apple Blue Mold

**Figure 2:**
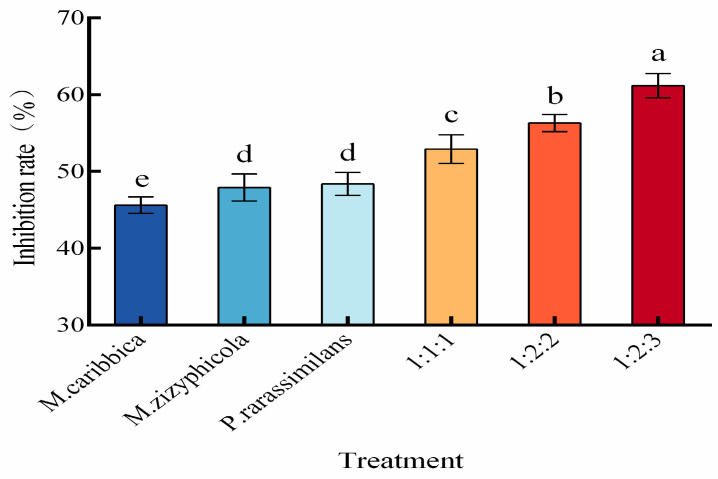
Biocontrol efficacy of single yeast strains and their mixtures against apple blue mold. Note: The data are means ± standard deviations. Different letters (a–e) indicate significant differences between groups (*p* < 0.05).

Based on preliminary screening, the ratio of the three yeast strains in mixed culture was optimized. Figure 2 shows that the inhibition rates against apple blue mold achieved by all mixed cultures (1:1:1, 1:2:2, and 1:2:3 volume ratios) were higher than those obtained with single-strain cultures. The mixing ratio of 1:2:3 yielded the highest biocontrol efficacy, suppressing disease development with an inhibition rate of 60.19%. This was significantly higher (*p* < 0.05) than that of other mixing ratios or individual yeast treatments.

### 3.3. Optimization of Nutrient Conditions for Mixed Culture

**Figure 3:**
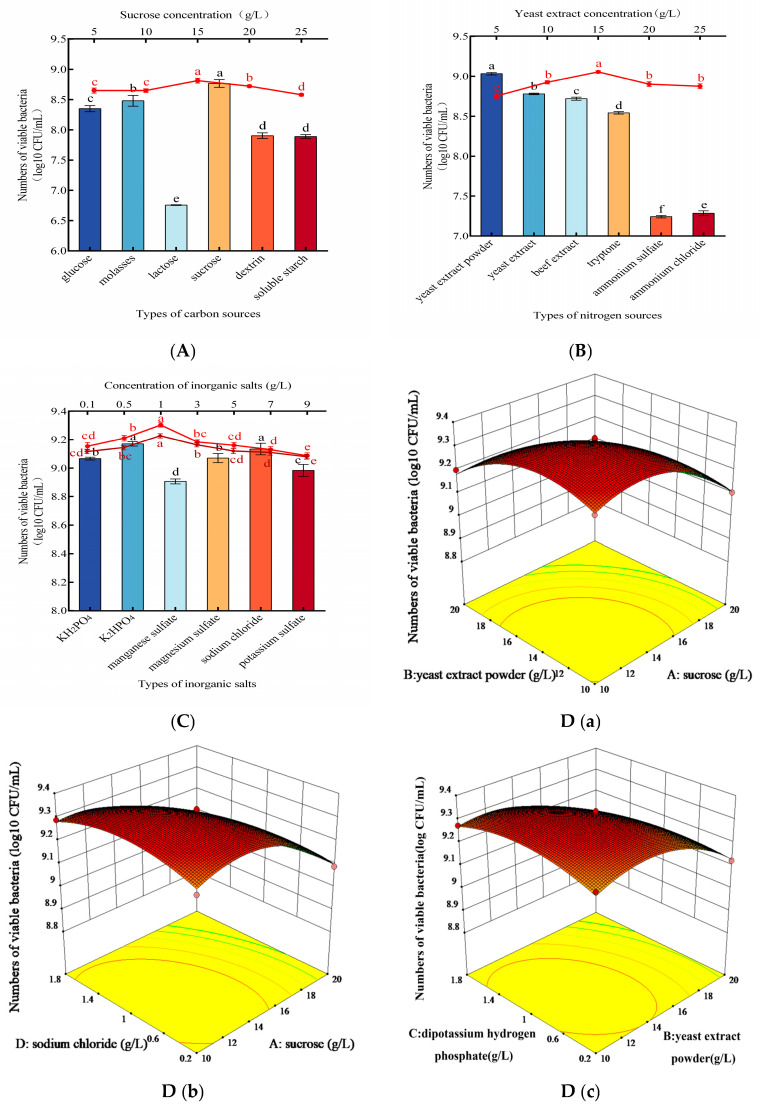

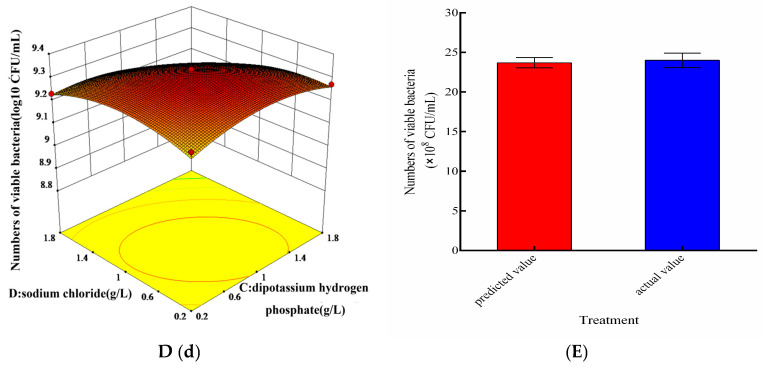
Optimization of protective additive formulation and model validation for the mixed yeast consortium. (**A**–**C**) Effects of carbon source, nitrogen source, and inorganic salt type and concentration on yeast growth, as determined by one-factor-at-a-time experiments. (**D**) Response surface methodology (RSM) plot showing the interaction between key factors. (**E**) Experimental validation of the predicted optimal model. Note: (**A**) Effects of carbon sources and optimal sucrose concentration. (**B**) Effects of nitrogen sources and optimal yeast extract concentration. (**C**) Effects of inorganic salts and optimal concentrations of K_2_HPO_4_ (red line) and NaCl (brown line). (**D**) Response surface plots showing the interactive effects: (**a**) sucrose and yeast extract powder, (**b**) sucrose and NaCl, (**c**) yeast extract powder and K_2_HPO_4_, (**d**) K_2_HPO_4_ and NaCl. The color gradient from green to red represents the increase in the response value. (**E**): Correlation between predicted and experimental values for model validation. Different letters (a–d) indicate significant differences between groups (*p* < 0.05).

Results from optimizing the nutrient conditions for the mixed yeast culture (1:2:3 ratio) are presented in Figure 3. Single-factor experiments were first conducted to identify the optimal components from six carbon sources, six nitrogen sources, and six inorganic salts. Their concentrations were then optimized. Among the carbon and nitrogen sources tested, sucrose (15 g/L) and yeast extract powder (15 g/L) yielded the highest viable cell counts (Figure 3A,B). Subsequent screening of inorganic salts identified K_2_HPO_4_ and NaCl as the most effective. The optimal concentrations for both K_2_HPO_4_ and NaCl were determined to be 1 g/L, producing the highest viable cell counts of 9.30 log10 CFU/mL and 9.23 log10 CFU/mL, respectively (Figure 3C). Thus, a composite inorganic salt supplement of K_2_HPO_4_ (1 g/L) and NaCl (1 g/L) was used in all subsequent experiments.

Based on the single-factor results, a four-factor, three-level Box–Behnken response surface methodology (RSM) design was implemented, and corresponding results are presented in Table S1. Analysis of variance (ANOVA) for the model is presented in Table 1. The response data were fitted by multiple linear regression and a second-order polynomial model, yielding the regression equation (Equation (1)):

Y = 9.32 − 0.13A − 0.096B − 0.025C − 0.042D − 0.042AB + 0.012AC − 0.047AD − 0.035BC − 0.016BD − 0.04CD − 0.12A^2^ − 0.1B^2^ − 0.057C^2^ − 0.061D^2^ (1)

where Y = viable cell count (log10 CFU/mL); A = sucrose concentration; B = yeast extract powder concentration; C = K_2_HPO_4_ concentration; D = NaCl concentration.

The response-surface plots (Figure 3D) reveal significant interactive effects on viable cell counts for the following pairs: yeast extract powder × sucrose, NaCl × sucrose, K_2_HPO_4_ × yeast extract powder, and NaCl × K_2_HPO_4_.

The regression model predicted that the optimal medium composition was sucrose 12.49 g/L, yeast extract powder 13.3 g/L, K_2_HPO_4_ 0.88 g/L, NaCl 0.95 g/L. Under optimized conditions, the maximum viable cell count was forecasted to be 2.37 × 10^9^ CFU/mL. Experimental verification under optimized conditions yielded a viable cell count of 2.40 × 10^9^ CFU/mL (Figure 3E), which differed from the predicted value by only 0.05%. This close agreement confirms the high accuracy and reliability of the model.

### 3.4. Effect of Cultivation Conditions on Mixed Culture

The effects of pH, inoculum concentration, incubation temperature, and incubation time on the viable cell count of the mixed culture grown in the optimized medium were investigated (Figure S1). The highest viable cell count (9.44 log10 CFU/mL) was achieved under the following conditions: pH 7.0, total inoculum concentration 1% (*v*/*v*), incubation temperature 24 °C, and incubation time 60 h.

### 3.5. Effect of Thickeners, Emulsifiers, and Antioxidants on Viable Cell Count of the Liquid Composite BCA

**Figure 4:**
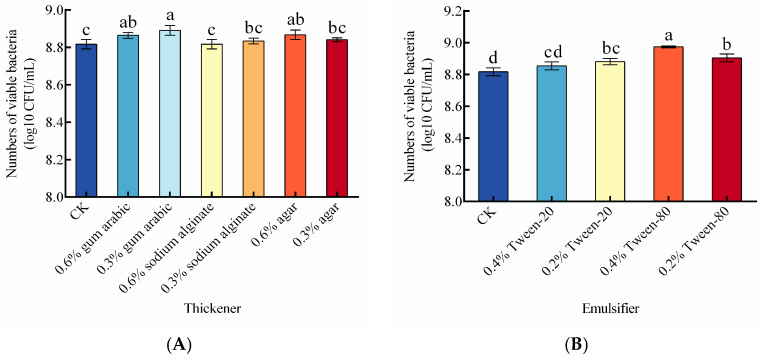

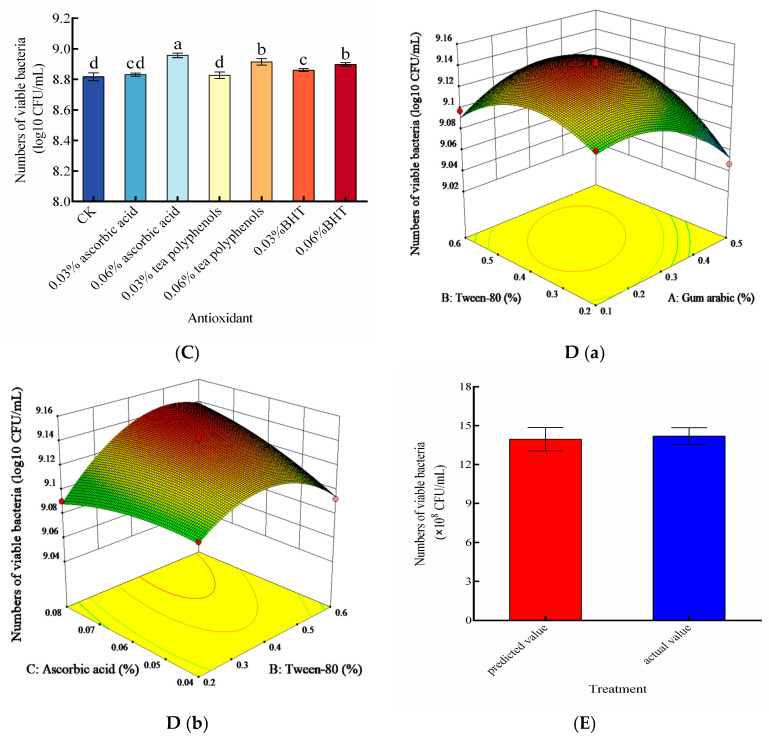
Optimization of protective additive formulation and model validation for the mixed yeast consortium. (**A**–**C**) Effects of the type and concentration of protective agents, emulsifiers, and antioxidants on the protective efficacy, as determined by one-factor-at-a-time experiments. (**D**) Response surface methodology (RSM) plot showing the interaction between key factors. (**E**) Experimental validation of the predicted optimal model. Note: Effects of different (**A**) thickeners, (**B**) emulsifiers, and (**C**) antioxidants of the Liquid Composite BCA on the viable cell count after 7 days of storage. (**D**) Response surface plots showing the interactive effects: (**a**) gum arabic and Tween-80; (**b**) Tween-80 and ascorbic acid. The color gradient from green to red represents the increase in the response value. (**E**) Correlation between predicted and experimental values for model validation. Different letters (a–d) indicate significant differences between groups (*p* < 0.05).

The influences of different protective additives on the viable cell count of the composite BCA after 7 days of storage are shown in Figure 4A–C. Single-factor experiments identified the optimal protective agents and their concentrations for maintaining high viable counts: 0.3% gum arabic (thickener), 0.4% Tween-80 (emulsifier), and 0.06% ascorbic acid (antioxidant).

Based on the single-factor results, a three-factor, three-level Box–Behnken RSM design was employed to optimize the concentrations of gum arabic (A), Tween-80 (B), and ascorbic acid (C) (Table S2). ANOVA for the model is presented in Table 2. According to F-values, the order of factors affecting viable cell counts was as follows: Tween-80 > gum arabic > ascorbic acid. The response data were fitted via multiple linear regression to a second-order polynomial model, yielding the regression equation (Equation (2)):

Y = 9.14 − 0.00875A + 0.011B + 0.0075C + 0.017AB − 0.0055AC + 0.014BC − 0.023A^2^ − 0.025B^2^ − 0.0064C^2^ (2)

where Y = viable cell count (log10 CFU/mL) after 7 days, A = gum arabic concentration; B = Tween-80 concentration; C = ascorbic acid concentration.

The response-surface plots (Figure 4D) reveal significant interactive effects on viable cell counts for the following pairs: Tween-80 × gum arabic, ascorbic acid × Tween-80.

The regression model predicted the optimal protective agent composition as: gum arabic 0.27%, Tween-80 0.49%, ascorbic acid 0.079%. Under these conditions, the predicted maximum viable cell count was 1.40 × 10^9^ CFU/mL. Experimental verification under optimized conditions yielded a viable cell count of 1.42 × 10^9^ log10 CFU/mL (Figure 4E), deviating by only 0.05% from the predicted value, which again demonstrates the high accuracy and reliability of the model.

### 3.6. Biocontrol Efficacy of the Composite BCA Against Blue Mold

**Figure 5:**
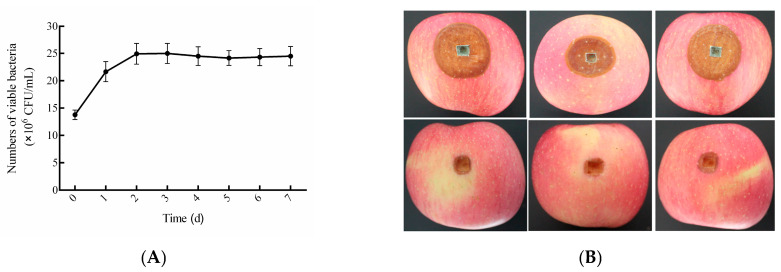

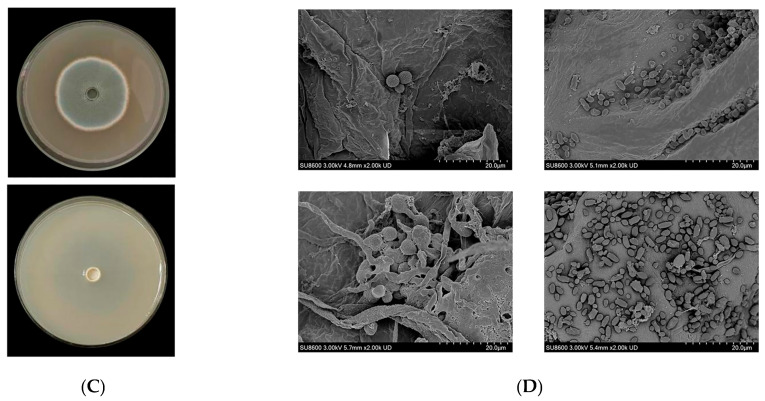
Biocontrol Efficacy of the Composite BCA against Blue Mold. Note: (**A**): Population dynamics of the BCA in apple wounds; (**B**): Effect of the BCA against *P. expansum* on apples (**Top**: Control group; **Bottom**: BCA-treated group); (**C**): Inhibitory effect of the BCA against *P. expansum* on PDA medium (**Top**: Control group; **Bottom**: BCA-treated group); (**D**): SEM observations of BCA suppression on *P. expansum* spore germination in apple wounds (**Top-left**: 24 h control; **Top-right**: 24 h BCA-treated; **Bottom-left**: 48 h control; **Bottom-right**: 48 h BCA-treated).

As shown in Figure 5A, the composite BCA exhibited excellent colonization ability on apple wounds. The yeast population rapidly colonized and proliferated in apple wounds, reaching the logarithmic growth phase within 2 days. Then, the yeast population stabilized and was maintained at approximately 2.53 × 10^7^ CFU/mL, indicating successful colonization and persistence at the wound site. SEM micrographs (Figure 5D) revealed pathogen-antagonist interaction dynamics. In the control group (no BCA), *P. expansum* conidia began to germinate sparsely 24 h post-inoculation (hpi). By 48 hpi, extensive hyphal growth with robust mycelial development was evident. In contrast, wounds treated with the composite BCA showed no spore germination at 24 hpi, and only minimal germ tube emergence was observed in a few spores even at 48 hpi, demonstrating a strong suppressive effect on pathogen germination and early growth. Apples treated with the composite BCA showed markedly smaller lesion diameters compared to the control group (Figure 5B), achieving an inhibition rate of 71.43% by day 6 post-inoculation. Similarly, the direct inhibitory effect of the BCA on *P. expansum* growth was evident on PDA plates (Figure 5C). After 6 days of incubation, *P. expansum* in the untreated control group formed colonies reaching 5 cm in diameter, whereas the treated group showed complete inhibition of fungal growth, demonstrating its potent in vitro antifungal activity. These results collectively confirm that the liquid composite BCA significantly suppresses *P. expansum* growth both in vivo on apple fruit and in vitro on artificial media.

### 3.7. Degradation of Patulin (PAT) by the Composite BCA in Fruit Juices

**Figure 6:**
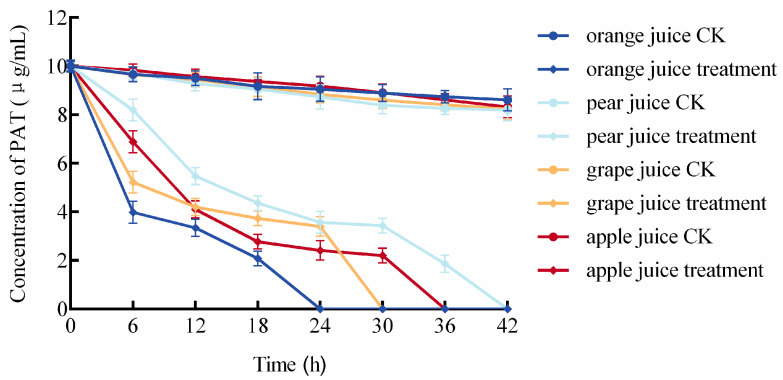
Patulin (PAT) degradation by the composite BCA in different fruit juices.

The patulin degradation efficacy of the composite biocontrol agent (BCA) in orange, pear, apple, and grape juices (spiked with 10 μg/mL PAT) is shown in Figure 6. Degradation rates differed among juice types. Notably, complete PAT degradation was achieved within 42 h in all tested juices. Degradation proceeded most rapidly in orange juice, with full degradation occurring within 24 h. Grape and apple juices followed, requiring 30 h and 36 h for complete degradation, respectively, whereas pear juice exhibited the slowest degradation, requiring 42 h for complete PAT degradation. These results demonstrate the robust PAT-degrading capacity of the composite BCA in diverse fruit juice systems.

### 3.8. Effect of the Composite BCA on Juice Quality

**Figure 7:**
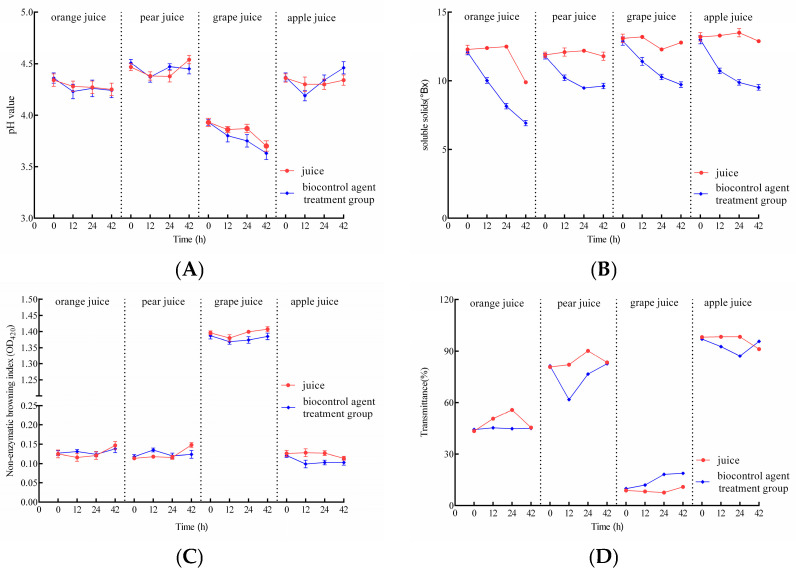
Effect of the Liquid Composite BCA on the quality of fruit juices. Note: (**A**): pH value; (**B**): soluble solids content (SSC); (**C**): non-enzymatic browning index (NEBI); (**D**): light transmittance.

The effect of adding the composite BCA on key juice quality parameters (pH, soluble solids content (SSC), non-enzymatic browning index (NEBI), and light transmittance) is presented in Figure 7. Overall, the addition of Composite BCA had little influence on the pH of any juice. SSC decreased significantly in all four juices, likely due to yeast utilizing a portion of the sugars. NEBI values remained nearly unchanged, indicating no adverse effect on this color-related parameter. Light transmittance values fluctuated slightly during the first 42 h but stabilized with minimal net change by the end of the incubation period. Notably, transmittance increased in grape juice after BCA treatment, suggesting a potential beneficial effect on juice clarity. Collectively, the composite BCA had only minor effects on the assessed juice quality parameters.

### 3.9. Storage Stability of the Liquid Composite BCA

**Figure 8:**
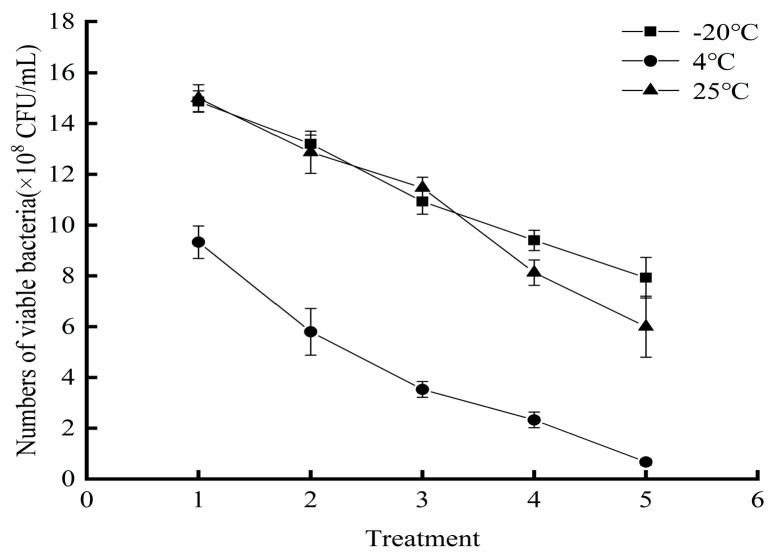
Viability of the liquid BCA at different temperatures. Note: 1: −20 °C and 4 °C for 30 d, 25 °C for 5 d; 2: −20 °C and 4 °C for 60 d, 25 °C for 10 d; 3: −20 °C and 4 °C for 90 d, 25 °C for 15 d; 4: −20 °C and 4 °C for 120 d, 25 °C for 20 d; 5: −20 °C and 4 °C for 150 d, 25 °C for 25 d.

Storage temperature critically influenced the viability of the liquid composite BCA. As quantified in Figure 8, viable cell counts progressively declined across all tested temperatures, with decline rates ordered as: −20 °C < 4 °C < 25 °C. Under ambient storage (25 °C), viability dropped to 6.0 × 10^8^ log10 CFU/mL within 1 month, representing a 57.7% reduction from the initial titer. Refrigerated storage (4 °C) provided intermediate stability, while −20 °C storage optimally preserved bioactivity, maintaining 7.93 × 10^8^ CFU/mL after 5 months. These results demonstrate that frozen storage at −20 °C effectively maintains the viability of the liquid composite BCA, confirming its optimal preservation efficacy for the liquid biocontrol agent.

## 4. Discussion

Antagonistic yeasts exert their biocontrol effects through four principal modes of action: competition for nutrients and space, secretion of antimicrobial compounds, mycoparasitism, and induction of host resistance [2]. Biocontrol efficacy varies significantly depending on the metabolic activity, life cycle, reproductive capacity, and environmental adaptability of individual yeast strains. In general, single-strain biocontrol agents (BCAs) often exhibit narrow antifungal spectra, limited modes of action, and suboptimal efficacy. Consequently, commercial yeast-based BCAs increasingly favor combinations of yeasts possessing either identical or complementary antagonistic mechanisms. This strategy enhances overall efficacy, reduces application frequency and dosage, and mitigates the risk of pathogen resistance development [26]. For instance, in studies on the biocontrol of *Penicillium digitatum* (citrus green mold) by yeast consortia, three yeast species all shared the ability to produce cell wall-degrading enzymes such as chitinase and protease, along with leucine arylamidase activity. Additionally, each species contributed unique mechanisms: *M. guilliermondii* exhibited high biofilm formation and produced antifungal volatile organic compounds (VOCs). *H. uvarum* secreted β-1,3-glucanase while producing mycocins to inhibit *P. digitatum* growth. *M.* aff. *pulcherrima* displayed the highest biocontrol activity due to its production of a broader enzyme repertoire. The synergistic action of the consortium significantly reduced lesion diameter and disease incidence, enabling sustainable control of *P. digitatum*-induced decay [18]. However, incompatibility between different antagonistic yeast species can compromise their combined efficacy and critically diminish the overall biocontrol effectiveness of the consortium. For example, the co-application of two *Rhodotorula glutinis* strains (SL1 and SL30) resulted in lower suppression efficiency against gray mold on apple fruit wounds compared to single-strain treatments [27]. This reduction is likely due to interspecific competition for nutrients and space among the yeasts, leading to an overall decrease in antimicrobial activity against the phytopathogen. Therefore, research on enhancing antimicrobial efficiency through yeast consortia should prioritize strain selection to avoid such antagonistic interactions, maximizing synergistic effects under conditions of coexistence within the same system. The absence of inhibition zones (Figure 1) in the cross-compatibility assay among *M. caribbica*, *M. zizyphicola*, and *P. rarassimilans* confirmed mutual biocompatibility, enabling their combined use. This compatibility underpinned the significantly higher biocontrol efficiency (71.43% inhibition rate against apple blue mold, Figure 2) achieved by the 1:2:3 (*v*/*v*/*v*) consortium compared to the single-strain treatments(*p* < 0.05). This is comparable to the synergistic effects for yeast combinations observed in the citrus study (78.40–83.18% mycelial growth inhibition) [18].

Compared with solid formulations, liquid microbial agents offer many advantages, such as simplified preparation, reduced costs, and rapid onset of action. However, their viability and stability are highly susceptible to environmental factors (temperature, light, oxygen), leading to cell aging and inactivation [28]. Maintaining high viable cell counts is paramount for the efficacy of liquid BCAs against fruit pathogens [29]. Therefore, we used viable cell count as the core metric in developing the composite biocontrol agent. This parameter is co-regulated by multiple factors: the genetic traits of constituent strains, the nutrient composition of the medium, the physical cultivation parameters, and the protective additives [30]. The intrinsic genetic characteristics of the strains determine the baseline activity of the consortium; Optimizing the medium’s nutrient profile (e.g., C/N ratio) and cultivation parameters (pH, temperature, etc.) enhances the yeast’s growth environment and directly maximizes proliferation efficiency, while protective additives mitigate viability decay by forming physicochemical barriers. By screening thickeners, emulsifiers, and antioxidants, we established a hurdle system to prevent viability decline and maintain formulation stability, thereby ensuring efficacy during storage and application [31,32,33]. All protective additives used were food-grade, and dual-response surface optimization enhanced rapid yeast proliferation while reducing viability decay in the composite biocontrol agent. The optimized agent colonizes apple wounds within 48 h (Figure 5A). Additionally, in vivo experiments on apple wounds and in vitro assays using culture media further validated the sustained pathogen suppression capability of the BCA(Figure 5C,D). After 5 months of storage at −20 °C, the viable count remained at 7.93 × 10^8^ CFU/mL, demonstrating excellent retention of activity.

Fruit intended for processing typically exhibits inferior quality and more severe mechanical damage than fresh-market fruit, increasing its susceptibility to pathogenic fungi and mycotoxin contamination. Global large-scale surveys have detected patulin (PAT) in apple, pear, hawthorn, orange, and grape juices, as well as jams [34,35,36]. The inherently acidic nature of fruit products, combined with PAT’s molecular stability under acidic conditions, poses significant challenges for effective PAT degradation in practical applications [37]. The composite biocontrol yeast formulation developed in this study significantly reduced patulin (PAT) levels in commercially available juices (10 μg/mL), with complete degradation achieved within 24 h in orange juice and up to 42 h in pear juice (Figure 6), aligning with findings by Ning et al. [38]. Yeast-mediated PAT reduction occurs through two primary pathways: adsorption and biodegradation. Current research has identified four PAT biodegradation products, all demonstrating lower or negligible toxicity compared to PAT [39]. Notably, the agent addition exerted minimal impact on key juice quality parameters: pH, non-enzymatic browning index (NEBI), and light transmittance (*p* > 0.05), although a significant decrease (*p* < 0.05) in soluble solids content (SSC) was observed, likely due to yeast sugar metabolism [40]. The non-enzymatic browning index (NEBI), a critical indicator for juice color [41,42], showed minor fluctuations but negligible overall change. Collectively, the agent exerted limited influence on overall juice quality. Subsequent work would shift from gross composition to sensory relevance: targeted profiling of volatile compounds and formal sensory panels will reveal whether the subtle metabolic activity detected here translates into perceptible differences for the consumer. Although this study demonstrated the high degradation efficiency of the biocontrol agents, the identity and toxicological status of the resulting products remain unknown. Therefore, future research will focus on characterizing these metabolites to ensure their safety and applicability.

## 5. Conclusions

**Figure 9:**
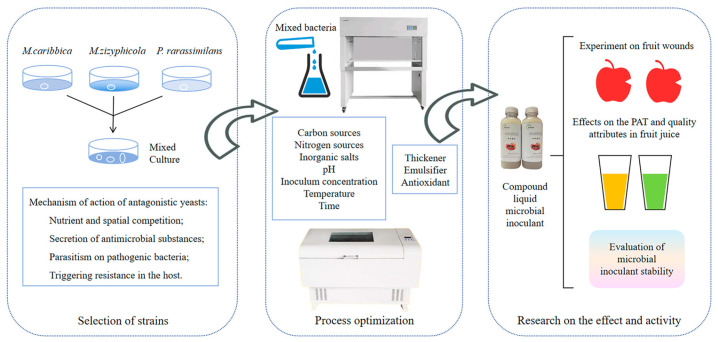
From Preparation to Application: The Composite BCA for Suppressing Blue Mold and Degrading Patulin.

In conclusion, this study screened and combined three antagonistic yeast strains (*M. caribbica*, *M. zizyphicola*, and *P. rarassimilans*) effective for the biocontrol of postharvest blue mold and patulin degradation in apples. The culture conditions and protective agent formulation were optimized using Response Surface Methodology (RSM). Optimal biomass production was achieved with a 1:2:3 (*v*/*v*/*v*) yeast ratio in medium containing sucrose (12.49 g/L), yeast extract powder (13.3 g/L), K_2_HPO_4_ (0.88 g/L), and NaCl (0.95 g/L) under pH 7.0, 1% total inoculum concentration, 24 °C, and a 60 h incubation. The liquid BCA formulation, stabilized with 0.27% gum arabic, 0.49% Tween-80, and 0.079% ascorbic acid, maintained high viability (9.15 log10 CFU/mL after 7 days). The high-efficacy composite biocontrol liquid agent that we developed overcomes the limitations of single-microbe preparations in control efficacy. The optimized formulation achieves rapid colonization at apple wound sites, effectively suppressing postharvest blue mold infections while degrading patulin in various fruit juices without compromising key juice quality parameters. By integrating multiple antagonistic yeasts into a functional microbial consortium, this work provides a green, economical, and efficient solution for designing tailored biocontrol agents (BCAs) targeting specific postharvest challenges (Figure 9), particularly disease suppression and mycotoxin detoxification in apples and other fruits.

## Figures and Tables

**Table 1:** Analysis of variance table for the regression equation in medium optimization.

Type	SS	DF	MS	F-Value	*p*-Value	Significance
Model	0.52	14	0.037	103.70	<0.0001	**
A	0.21	1	0.21	594.67	<0.0001	**
B	0.11	1	0.11	308.60	<0.0001	**
C	0.0075	1	0.0075	21.04	0.0004	**
D	0.021	1	0.021	57.97	<0.0001	**
AB	0.007	1	0.007	19.79	0.0006	**
AC	0.0006	1	0.0006	1.68	0.2154	
AD	0.0088	1	0.0088	24.79	0.0002	**
BC	0.00497	1	0.00497	13.94	0.0022	**
BD	0.00096	1	0.00096	2.70	0.1229	
CD	0.0062	1	0.00624	17.51	0.0009	**
A^2^	0.090	1	0.090	251.81	<0.0001	**
B^2^	0.066	1	0.066	186.13	<0.0001	**
C^2^	0.021	1	0.021	58.63	<0.0001	**
D^2^	0.024	1	0.024	67.74	<0.0001	**
Residual	0.00499	14	0.00035			
Lack of fit	0.00465	10	0.00046	5.53	0.0569	Not significant
Pure error	0.00033	4	0.000084			

Note: **, Significant at *p* < 0.01.

**Table 2:** Analysis of variance table for the regression equation of protective agent.

Type	SS	DF	MS	F-Value	*p*-Value	Significance
Model	0.00956	9	0.00106	25.02	0.0002	**
A	0.00061	1	0.00061	14.42	0.0067	**
B	0.00088	1	0.00088	20.77	0.0026	**
C	0.00045	1	0.00045	10.60	0.0140	*
AB	0.00122	1	0.00122	28.84	0.0010	**
AC	0.00012	1	0.00012	2.85	0.1353	
BC	0.00084	1	0.00084	19.80	0.0030	**
A^2^	0.00220	1	0.00220	51.99	0.0002	**
B^2^	0.00261	1	0.00261	61.47	0.0001	**
C^2^	0.00017	1	0.00017	4.06	0.0837	
Residual	0.00029	7	0.00004			
Lack of fit	0.0002	3	0.00006	2.76	0.1757	Not significant
Pure error	0.00009	4	0.00002			

Note: **, * Significant at *p* < 0.01 and *p* < 0.05, respectively.

## Data Availability

The original contributions presented in the study are included in the article, further inquiries can be directed to the corresponding author.

## Supplementary Materials

The following supporting information can be downloaded at: https://www.mdpi.com/article/10.3390/foods14193378/s1, Figure S1: Effect of cultivation conditions on mixed culture; Table S1: Response surface methodology design and experimental results for medium optimization; Table S2: Response surface methodology design and experimental results for protective additives optimization.

## Abbreviations

The following abbreviations are used in this manuscript:

BCA	Biocontrol agent
PAT	Patulin
RSM	Response surface methodology
HPLC	High-performance liquid chromatography
NYDA	Nutrient yeast dextrose agar
NYDB	Nutrient yeast dextrose broth
BHT	Butylated hydroxytoluene
SEM	Scanning electron microscopy
SSC	Soluble solids content
NEBI	Non-enzymatic browning index
ANOVA	Analysis of variance
VOCs	Volatile organic compounds
