# Supplementary material for: Construction of Composite Biocontrol Agent (BCA): Developing Effective Strategies for Controlling Postharvest Blue Mold and Patulin in Apples
Source: Foods. 2025 Sep 29;14(19):3378. doi: 10.3390/foods14193378 (PMC12523723; doi:10.3390/foods14193378)
Supplement: Supplementary file 1 [file foods-14-03378-s001.zip › foods-3882474-supplementary.pdf]

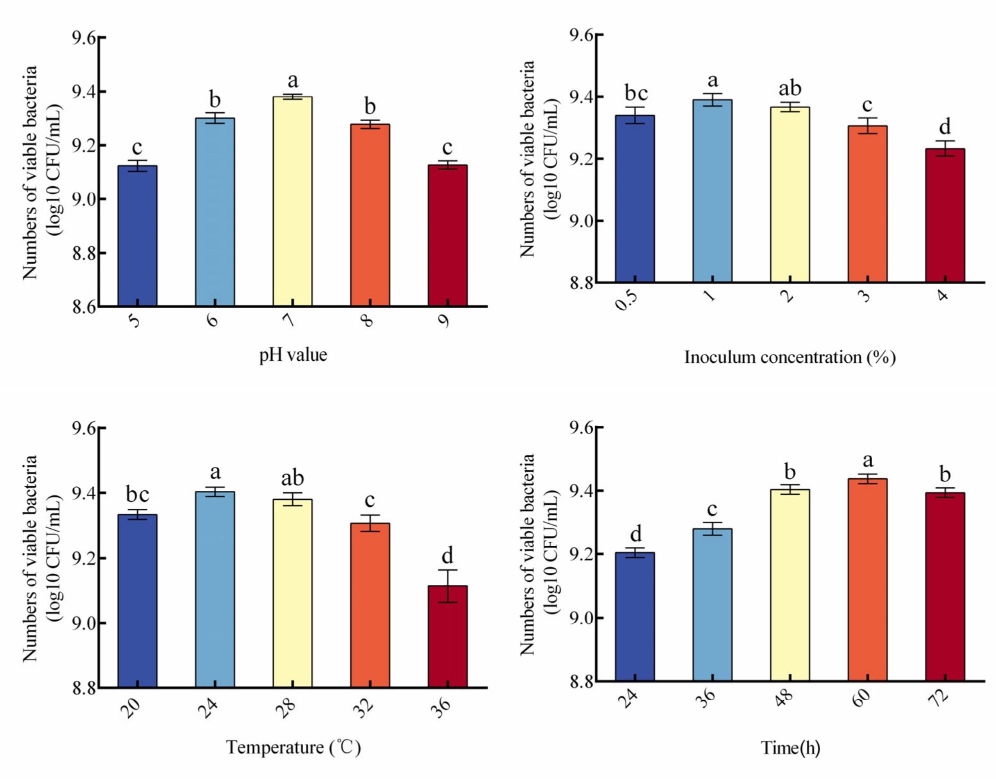

Figure S1. Effect of cultivation conditions on mixed culture.

Table S1. Response surface methodology design and experimental results for medium optimization.

Experimental number	A	B	C	D	Viable cell count (lg cfu/mL)
1	10	10	1	1	9.285
2	20	10	1	1	9.106
3	10	20	1	1	9.201
4	20	20	1	1	8.854
5	15	15	0.2	0.2	9.258
6	15	15	1.8	0.2	9.272
7	15	15	0.2	1.8	9.232
8	15	15	1.8	1.8	9.088
9	10	15	1	0.2	9.249
10	20	15	1	0.2	9.091
11	10	15	1	1.8	9.289
12	20	15	1	1.8	8.943
13	15	10	0.2	1	9.266
14	15	20	0.2	1	9.124
15	15	10	1.8	1	9.274
16	15	20	1.8	1	8.991
17	10	15	0.2	1	9.308
18	20	15	0.2	1	9.001

19	10	15	1.8	1	9.261
20	20	15	1.8	1	9.003
21	15	10	1	0.2	9.282
22	15	20	1	0.2	9.119
23	15	10	1	1.8	9.223
24	15	20	1	1.8	8.998
25	15	15	1	1	9.329
26	15	15	1	1	9.323
27	15	15	1	1	9.312
28	15	15	1	1	9.317
29	15	15	1	1	9.335

Table S2. Response surface methodology design and experimental results for protective additive optimization.

Experimental number	A	B	C	Viable cell count (lg cfu/mL)
1	0.1	0.2	0.06	9.109
2	0.5	0.2	0.06	9.047
3	0.1	0.6	0.06	9.098
4	0.5	0.6	0.06	9.106
5	0.1	0.4	0.04	9.100
6	0.5	0.4	0.04	9.103
7	0.1	0.4	0.08	9.125
8	0.5	0.4	0.08	9.106
9	0.3	0.2	0.04	9.104
10	0.3	0.6	0.04	9.093
11	0.3	0.2	0.08	9.091
12	0.3	0.6	0.08	9.138
13	0.3	0.4	0.06	9.134
14	0.3	0.4	0.06	9.144
15	0.3	0.4	0.06	9.133
16	0.3	0.4	0.06	9.142
17	0.3	0.4	0.06	9.136
